# Acquired immunodeficiency syndrome-related acute longitudinal myelitis involving the entire spinal cord

**DOI:** 10.1007/s10072-022-06034-7

**Published:** 2022-03-30

**Authors:** Yu Zhao, Fuxiang Wang, Liming Cao

**Affiliations:** 1 Department of Neurology, Shenzhen Third People’s Hospital, Shenzhen, 518000 China; 2 Department of Neurology, The Second Affiliated Hospital, Southern University of Science and Technology, Shenzhen, 518000 China; 3 Department of Infectious Diseases, Shenzhen Third People’s Hospital, Shenzhen, 518000 China; 4 Department of Infectious Diseases, The Second Affiliated Hospital, Southern University of Science and Technology, Shenzhen, 518000 China; 5 Department of Neurology, The First Affiliated Hospital of Shenzhen University, 3002 Sungang West Road, Futian District, Shenzhen, 518000 China

**Keywords:** Acquired immunodeficiency syndrome, Human immunodeficiency virus, Immunotherapy, Longitudinal myelitis, Magnetic resonance imaging

## Abstract

**Introduction:**

As acquired immunodeficiency syndrome (AIDS) becomes more widespread, there will be an increasing need for diagnostic AIDS-related neurological syndromes. AIDS-related myelitis is easy to be ignored, and AIDS-related longitudinal myelitis has not yet been reported.

**Case presentation:**

A 45-year-old male patient was admitted to our hospital after 3 days of progressive slurred speech and limb weakness. Neurologic examination revealed near-complete four-limb paralysis with dyspnea, dysarthria, and neck rigidity. Contrast-enhanced T2-weighted magnetic resonance imaging showed hyperintensities within the entire spinal cord. Cerebrospinal fluid analysis showed elevated white blood cell count and protein level. He was administered high-dose immunoglobulin and methylprednisolone. There was rapid regression in his symptoms after a month of therapy.

**Conclusions:**

This unique presentation of AIDS with longitudinal myelitis involving the entire spinal cord enriches our understanding of the clinical spectrum of this condition. Our case provides essential information for the diagnosis and treatment of longitudinal myelitis in AIDS patients.

A 45-year-old man was admitted to our hospital after 3 days of progressive slurred speech and limb weakness accompanied by constipation and urinary retention. He was diagnosed with acquired immunodeficiency syndrome (AIDS) 3 years before presentation by an infectious disease specialist according to the WHO diagnostic criteria [1]. He was initially administered highly active antiviral therapy (oral tenofovir, lamivudine, and efavirenz) under the specialist’s guidance and then gradually developed rash and abnormal liver function related to efavirenz and drug resistance. Therefore, efavirenz was replaced with lopinavir-ritonavir from 2020, and the other antiviral drugs were retained. There was no history of genetic diseases, recent vaccinations, joint pain, or oral ulcers. Physical examination revealed somnolence, dyspnea with hypoxemia, dysarthria, neck rigidity, right hemilateral tactile hypoesthesia, and a significant decrease in muscle strength of 0/5 and 2/5 in the bilateral upper and lower limbs, respectively.

Laboratory studies showed elevated neutrophil percentage (93.5%), erythrocyte sedimentation rate (79 mm/h), and elevated levels of C-reactive protein (129.53 mg/L), serum aspergillus antigen (1.72), β-D glucan (247.65 pg/mL), and anti-SS-antigen-A/Ro52KD antibodies (173 AU/mL). Serum T-lymphocyte subset analysis showed that T lymphocyte (33.8%), T-helper lymphocyte (7.5%), and CD3 + CD4 + /CD3 + CD8 + (0.33) levels were decreased. Toluidine red unheated serum and cerebrospinal fluid (CSF) tests were positive. Syphilis antibodies were found in serum and CSF samples. Quantification of serum human immunodeficiency virus type 1 RNA using polymerase chain reaction showed an increased RNA level (1070 IU/mL; reference range: < 500 IU/mL); however, CSF human immunodeficiency virus RNA was undetectable. CSF analysis showed elevated leukocyte count (333 × 10^6^/L), protein level (65.18 g/L), and glucose level (6.26 mmol/L). His CSF and blood cultures yielded no bacterial, anaerobic, or fungal growth. There were no immunoglobulin M antibodies in the CSF for toxoplasma, rubella virus, cytomegalovirus, or herpes simplex virus. Oligoclonal bands are detected by separation of CSF proteins while not demonstrable in the serum. Magnetic resonance imaging showed hyperintense lesions in the central medulla oblongata (Fig. 1A-C), inferior-middle part of pons (Fig. 1C), and left ventriculus lateralis (Fig. 1D). Spinal magnetic resonance images showed hyperintensities within the entire spinal cord on T2-weighted imaging (Fig. 2A-K). Chest computed tomography showed infection in the left lower lobe of the lungs. The patient was initially treated with intravenous immunoglobulin (20 g/d) and intravenous methylprednisolone (0.12 g/d) for 4 days, followed by gradual dosage reduction until day 25. Other treatments included antiviral, antibiotic, and empirical antituberculous drugs. His dyspnea, dysarthria, and limb weakness were obviously improved. On day 10, he showed muscle strength on the left upper limb (4 + /5), right upper limb (3 − /5), and bilateral lower limbs (4 + /5). Unfortunately, the patient exhibited weakness and paralysis and recurrent dyspnea on follow-up. The patient refused ventilator-assisted breathing and abandoned therapy. He died at home 2 months after discharge.

**Fig. 1:**
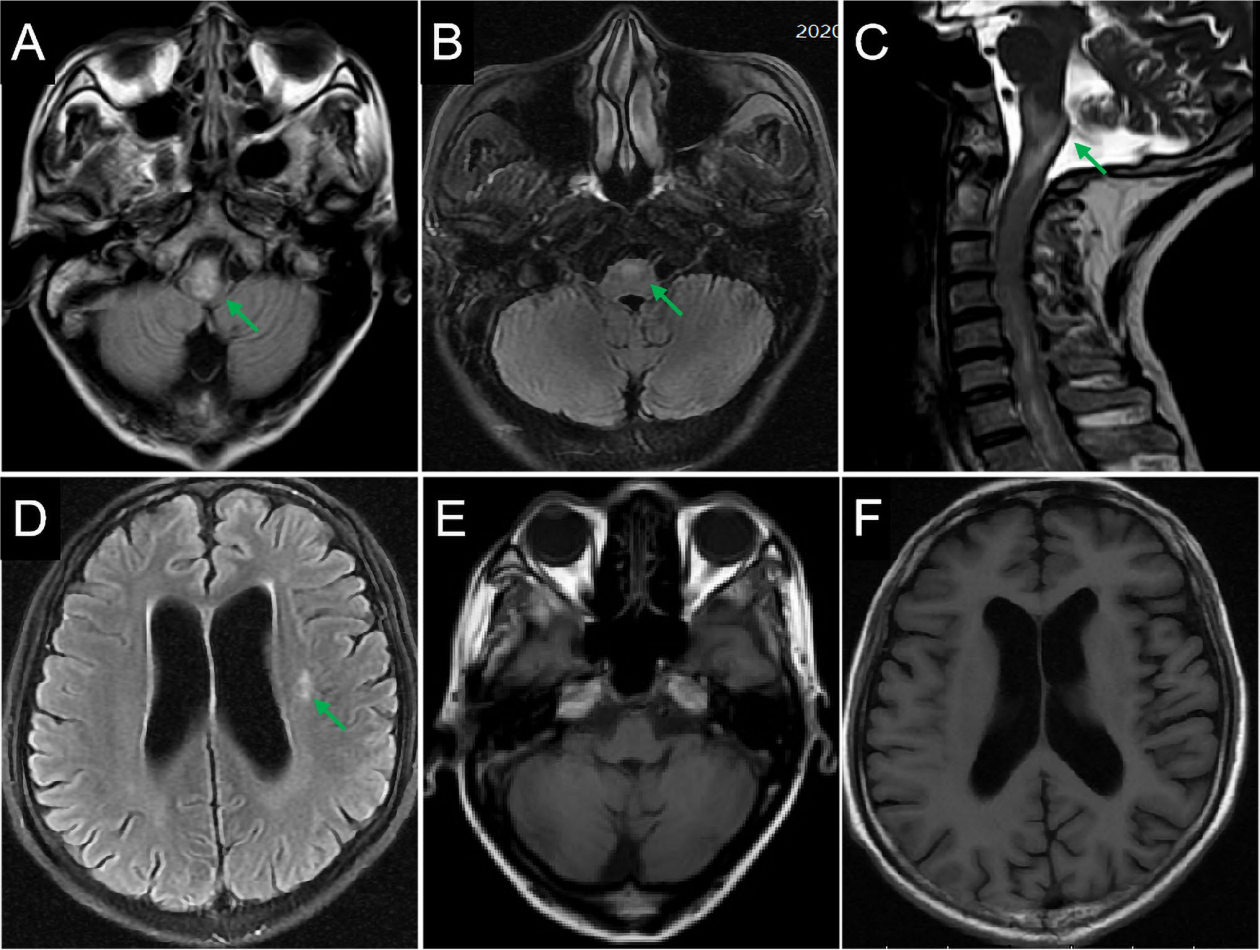
Brain magnetic resonance imaging. T2-weighted imaging (WI) showing hyperintense lesions in the central medulla oblongata (**A**, **B**, **C**, arrows), inferior-middle part of pons (**C**, arrow), and left ventriculus lateralis (**D**, arrow). There was hypointensity (**E**, **F**) in the corresponding part on T1-WI imaging

**Fig. 2:**
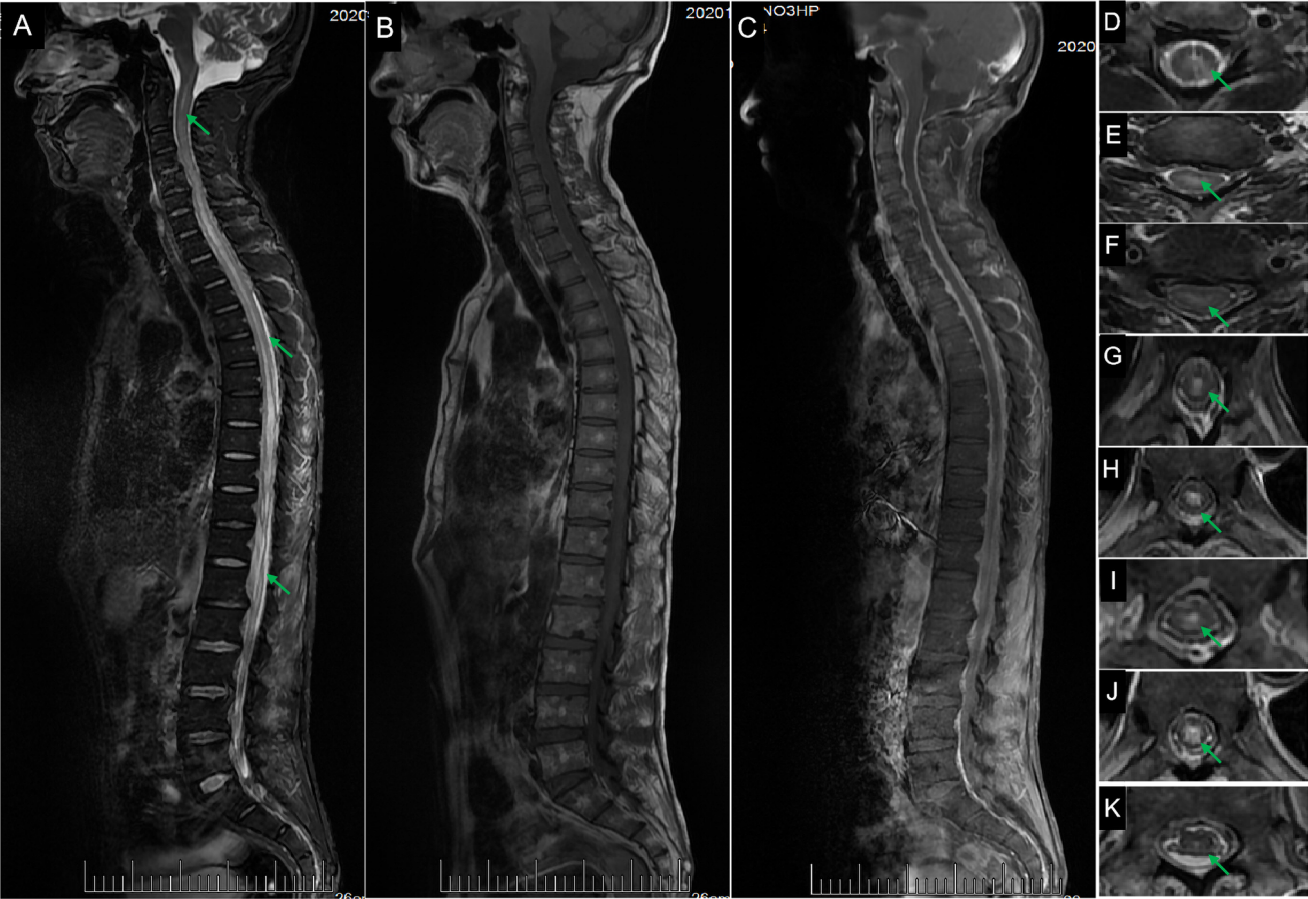
Spinal cord magnetic resonance imaging. Spinal cord magnetic resonance imaging shows hyperintensities (**A**, arrows) within the cervical, thoracic, and lumbar spinal cord on T2-weighted imaging (WI), and the corresponding lesions are hypointense (**B**) on T1-WI. Lesions are not enhanced on gadolinium-enhanced imaging (**C**). Axial T2-WI imaging shows intramedullary hyperintensity and edema, involving mainly the central part and sparing the peripheral spinal cord at different levels (**C**–**J**)

Acute longitudinal myelitis (LM) involves more than four spinal cord segments and is a rare comorbidity of AIDS. LM is likely to result in severe and disabling sequelae. LM may be caused by systemic lupus erythematosus [2], Japanese encephalitis, coronavirus disease 2019 [3], or Behçet’s syndrome [4]. To our knowledge, AIDS-related LM involving the entire spine has not yet been reported.

The findings extend the neuroimaging spectrum of AIDS-related LM. The patient’s symptoms improved rapidly after active immunotherapy. We considered that AIDS-related LM might be related to dysimmunity. There are no reports on AIDS-related LM treatment protocols and regimens. With our experience and the related reports [2–4], we recommend treatment with intravenous immunoglobulin methylprednisolone.

This case enriches our understanding of the treatment and neuroimaging spectrum of LM and AIDS-related central nerve injuries, specifically AIDS-related LM. A more comprehensive understanding of the etiology of LM is needed.

## Data Availability

Data sharing is not applicable to this article as no datasets were generated or analyzed during the current study.
